# Patterns of leisure participation among older people and their health conditions: a survey in Fujian, China

**DOI:** 10.3389/fpubh.2025.1492342

**Published:** 2025-07-21

**Authors:** Zhen Li, Xiao Yan

**Affiliations:** Shanghai Customs College, Shanghai, China

**Keywords:** leisure activity participation, older adults, health condition, successful aging, urban–rural differences

## Abstract

**Objective:**

This study aimed to explore the association between leisure activity participation and health conditions among older adults in Fujian Province, China.

**Methods:**

This is a population-based cross-sectional study including a multiple-stage sample of 527 subjects aged 60 years and older. Physical activity was evaluated using the long version of a questionnaire designed to assess the living conditions of Chinese older adults in urban and rural areas, using home interviews with a last-week recall. The questionnaire includes 14 different activities, which are grouped into 5 activity types.

**Results:**

Nearly 20% of the subjects participated in one or fewer leisure activities, and the young-old subjects and men were more likely to engage in vigorous activities than their counterparts. Older adults who had better self-rated health enjoyed more leisure activities than those who rated themselves in poor health after controlling for some demographic factors. Participants living in urban areas with better self-rated health and higher educational levels showed more participation in mental, physical, and productive activities.

**Conclusion:**

The findings identify significant correlates of leisure activity participation among older Chinese adults, providing preliminary evidence to support the development of successful aging initiatives and health policies for older populations.

## 1 Introduction

Increasing life expectancy, with decreasing infant mortality and fertility rates, has resulted in a rapid aging population in Fujian Province, China, where it is expected that the number of people aged 60 years and older has amounted to 16.86% of the total population in 2022, according to Fujian Statistics 2023 (1). The growing older population presents various challenges for families and society, with one of the most important being how these older people lead healthy and high-quality lives. Physical inactivity has been identified as the fourth leading risk factor for global mortality (6% of deaths globally) (2). It is not surprising that greater engagement in leisure activities is associated with a lower risk of stroke (3, 4), diabetes (5, 6), depressive symptoms and dementia (7, 8), psychological distress (9), and cancer mortality (10). Therefore, participation in activities is considered a critical pathway to successful aging (11), as it improves quality of life and increases longevity.

Previous studies have found evidence suggesting that health conditions are associated with leisure activity participation of older adults (12–14). For instance, chronic diseases are relevant factors that contribute to lower levels of activity participation (15). On the other hand, active individuals are less likely to develop several chronic diseases (16, 17). Physical inactivity is frequent in older adults with osteoarthrosis, diabetes, obesity, and cancer (18–20). Another cross-sectional study conducted in Malaysia found that older adults with poor glycemic control had a lower physical activity level (21).

As to subjective health, some studies have shown that good self-rated health has a consistent protective effect on leisure activity participants (22–24). According to a study conducted in Poland (25), bad health status is the most frequent barrier to participating in sports and physical activities. A study in Sweden found that health status was the strongest predictor of low physical activity (26).

Existing evidence predominantly examines leisure-health associations through single health lenses: chronic diseases (27–29) or self-rated health only (30). Few studies have explored the types of activities that individuals with both self-rated health and chronic diseases participate in. The majority of these studies were conducted in high-income countries, which had different activity patterns than low- and middle-income countries (31, 32). The activities that older adults engage in during their leisure time, their level of physical activity, the most popular activity among them, and how diseases and self-rated health affect their participation are all unknown. Current evidence on older people’s leisure activity participation in Fujian province, China, is limited. Thus, our study was motivated by the availability of a standardized questionnaire to describe leisure activity participation, as well as a paucity of studies addressing the five components of leisure activities, particularly in China. This study aimed to assess the relationship between types of leisure activity participation and different health aspects among older adults in Fujian Province, with a focus on self-rated health and chronic diseases. The hypothesis examines the potential associations between older adults’ participation in leisure activities and health-related factors.

## 2 Data and method

### 2.1 Study population

In August 2015, a cross-sectional investigation into the living conditions of Chinese older adults in urban and rural areas was conducted in Fujian Province, which is the most extensive investigation in the whole country regarding the situation of older adults. A total of 11 counties were selected without replacement, and the remaining counties were selected with a probability proportional to their size. From each selected county, four towns (street blocks) were randomly selected. In each selected town, 30 residents aged 60 years and older were chosen by simple random sampling. Of the 5,280 residents who received questionnaires, 9.98% (*n* = 527) responded to the long version, 96.77% (*n* = 510) of whom provided complete responses. This study only analyzes the data of residents who provided complete responses, aged 60–95 years old (*M* = 69.91, SD = 8.20). Among them, there were 236 men (46.27%) and 274 women (53.73%). Participants aged 60–69 years, 70–79 years, and 80 years and above accounted for 58.24, 24.90, and 16.86%, respectively. In terms of place of residence, urban household registration accounted for 47.84% and rural household registration accounted for 52.16%. In terms of education level, no formal education accounted for 25.10%, and middle school and higher accounted for 41.76%. Married participants accounted for 73.92%, with widowed/divorced/single participants accounting for 26.08%. The proportion of participants who rated their health as good was 36.67%, while the proportion of participants who rated their health as bad was 17.84%. Participants with no chronic diseases accounted for 24.90%, whereas those with two or more chronic diseases accounted for 42.55%.

### 2.2 Definition of variables

The dependent variable was the participation in leisure activities. The assessment of leisure activities was based on 15 dichotomous (yes or no) questions (translated from Chinese) on different activities, on which they participated on a regular basis. Fourteen different activities were grouped into five activity types, using the classification method adopted in previous studies (33). Reading books/newspapers and participating a college/institute for senior citizens constitute mental activity. Physical activities include walking/jogging, Tai Chi/exercises, dancing, and playing gate ball/pingpong/tennis. Social activities comprises going to the theater/cinema, playing mahjong/cards/chess, and attending social groups. Productive activities include gardening, keeping pets, and fishing/painting/photography/collecting. Creational activities include watching TV/listening to the radio and surfing the internet.

Self-rated health was used as an independent variable in this analysis. The subjects were asked the following question (translated from Chinese): How do you feel about your health? The self-reported health status was grouped into three types based on five choices (very good/good, average, and poor/very poor).

Chronic diseases were defined as the presence of any of the following 11 self-reported kinds of conditions: cataracts/glaucoma, hypertension, diabetes, cardiovascular diseases (CCVd; including coronary heart disease, angina, stroke), gastritis, osteoarthrosis (osteoporosis, arthritis, rheumatism, intervertebral disk disease [IVDD]), chronic lung disease (chronic obstructive pulmonary disease, tracheitis, and pulmonary emphysema), asthma, malignancy, diseases in the reproductive system, and other chronic conditions.

Regarding controlled variables, data regarding age and gender were collected directly from the questionnaire. Education was grouped into three education levels (no formal education, primary school, middle school, and higher) according to the subjects’ answer to the highest level of schooling. Living arrangements were categorized based on whether older adults lived alone or with some companions, including a partner, children, and other caregivers. In terms of marital status, older adults with a partner were defined as married, while those who were widowed, divorced, or never married were labeled as single.

### 2.3 Statistical analysis

The data were managed and analyzed using Stata (version 12.0). Descriptive statistics were used to show the distribution of older people’s participation in the 14 leisure activities based on their age group and gender. Descriptive statistics (means ± standard deviations) were used to compare activity frequencies across leisure groups. Between-group differences were evaluated using independent samples *t*-tests (for two groups) or one-way ANOVA (for ≥3 groups). The categorical variables were analyzed using *χ*^2^ tests. Logistic multi-nominal regressions were performed to analyze the factors associated with overall leisure activity participation, as well as the participation in mental, physical, social, productive, and recreational activities, with odds ratio and 95% confidence intervals. The research protocol was approved by the Institutional Review Board of Shanghai Customs College, and informed consent was obtained from all participants during the questionnaire survey, with procedures overseen by the China National Committee on Aging.

## 3 Results

### 3.1 Characteristics of the sample

Table 1 shows the engagement in each type of leisure activity as perceived by age and gender. In general, watching TV/listening to the radio was the most prevalent activity among men and women of all ages. As expected, younger old groups (60–69 years) were more active than older groups. The former prefer physical activities (e.g., walking/jogging, dancing, and playing gate ball/Ping-Pong/tennis), and their participation dropped with increasing age, while activities such as participating in college/institute for senior citizens and attending social groups were much more prevalent among the older group. Dancing is more popular among women than reading books/newspapers, playing gate ball /Ping-Pong /tennis, playing mahjong/cards/chess, attending social groups, fishing /painting /photography /collecting, and surfing the internet with men.

**Table 1:** Leisure activity participation rates (%) by age group and gender.

Leisure activity	Age group			Gender	
	60–69	70–79	80+	Female	Male
	*n* = 297	*n* = 127	*n* = 86	*n* = 274	*n* = 236
Reading books/newspapers	41.75	37.80	26.74	27.01	51.27
Participating in college/institute for senior citizens	6.73	6.30	8.14	6.20	7.63
Walking/jogging	62.63	54.33	41.86	56.57	57.63
Tai Chi/exercises	4.38	7.09	3.49	5.84	3.81
Dancing	10.10	3.94	0.00	9.49	3.81
Playing gate ball/Ping-Pong/tennis	2.69	1.57	1.16	0.00	4.66
Going to the theater/cinema	6.06	9.45	3.49	6.93	5.93
Playing mahjong/cards/chess	15.49	14.96	9.30	10.58	18.64
Attending social groups	34.68	33.86	37.21	30.66	39.83
Gardening	29.97	27.56	11.63	26.64	25.85
Keeping pets	4.71	4.72	3.49	5.47	3.39
Fishing/painting/photography/collecting	1.35	4.72	1.16	0.73	3.81
Watching TV/listening to the radio	97.31	96.85	90.70	94.53	97.88
Surfing the internet	15.15	5.51	6.98	8.39	14.83

Percentages are calculated within each age/gender subgroup.

Table 2 shows the older people participating in activities by activity type. The most popular leisure activities were walking/jogging and watching TV/ listening to the radio, which belonged to physical activity and recreational activity. All three were chosen by more than 50% of the subjects. The most popular activity type was recreational activity, while the least popular was mental activity.

**Table 2:** Total number and percentage of subjects participating in different leisure activities.

Activity type	1 ~ 5%	5 ~ 10%	≥10%	Total
Mental activity	-	Participating in college/institute for senior citizens	Reading books/newspapers^a^	230
Physical activity	Tai Chi/exercises; playing gate ball/Ping-Pong/tennis	Dancing	walking/jogging^b^	362
Social activity	-	going to the theater/cinema	playing mahjong/cards/chess; attending social groups^a^	284
Productive activity	Keeping pets; fishing/painting/photography/collecting	-	Gardening	168
Recreational activity	-	-	Surfing the internet; watching TV/ listening to the radio^b^	548

≥30%^a^; ≥50%^b^.

Table 3 shows the pattern of participation in leisure activities based on health indicators. Specifically, 19.02% of participants participated in one or fewer leisure activities, while 45.49% participated in two or three activities. Young-old participants and men were more likely to participate in vigorous activities than their counterparts. Consistent with Table 3, 31.02% of female participants engaged in four or more activities, while 24.09% reported one or fewer leisure activities. Subjects with higher education, married, and living with someone engaged in a larger number of activities. Among people with poor self-rated health, nine-tenths of the subjects were inactive. Participants with two or more chronic diseases had similar rates of participation in four or more leisure activities (34.56%) to those without chronic diseases (31.5%, *p* > 0.05). The mean number of activities in the study population was 3.12 ± 1.82, which decreased with age. The young-old (aged 60–69 years) were the most active, participating in an average of 3.33 activities, while the older participants were less engaged (3.09 activities for those aged 70–79 years old and 2.45 for those aged 80 + years). Subjects with a higher educational level reported more than four activities, while those with elementary education (2.58) or no formal education (2.13) reported fewer activities. In general, participants with bad self-rated health were less active (2.10), whereas those with good self-rated health were the most active (3.57). High variability within gender, age, education, groups, marital status, and self-rated health factors was observed.

**Table 3:** Demographic characteristics by leisure activity participation levels (values in parentheses represent percentages of participants within each subgroup).

Variable and category	*n* (±SD)	≤1 activity (%)	2–3 activities (%)	≥4 activities (%)	*χ*^2^
Age					*p* = 0.014
60–69	297 (3.33 ± 1.87)	48 (16.16)	130 (43.77)	119 (40.07)	
70–79	127 (3.09 ± 1.73)	24 (18.90)	60 (47.24)	43 (33.86)	
80+	86 (2.45 ± 1.60)	25 (29.07)	42 (48.84)	19 (22.09)	
Gender					*p* = 0.003
Female	274 (2.89 ± 1.78)	66 (24.09)	123 (44.89)	85 (31.02)	
Male	236 (3.39 ± 1.82)	31 (13.14)	109 (46.19)	96 (40.68)	
Geographic region					*p* < 0.001
Urban region	244 (3.78 ± 1.89)	25 (10.25)	91 (37.30)	128 (52.46)	
Rural region	266 (2.52 ± 1.51)	72 (27.07)	141 (53.01)	53 (19.92)	
Education					*p* < 0.001
No formal education	128 (2.13 ± 1.33)	47 (36.72)	63 (49.22)	18 (14.06)	
Primary school	169 (2.58 ± 1.36)	36 (21.30)	95 (56.21)	38 (22.49)	
Middle school and higher	213 (4.15 ± 1.87)	14 (6.57)	74 (34.74)	125 (58.69)	
Marital status					*p* = 0.001
Married	377 (3.31 ± 1.82)	60 (15.92)	168 (44.56)	149 (39.52)	
Widowed/divorced/ single	133 (2.58 ± 1.69)	37 (27.82)	64 (48.12)	32 (24.06)	
Living arrangement					*p >* 0.05
With someone	448 (3.16 ± 1.83)	84 (18.75)	201 (44.87)	163 (36.38)	
Alone	62 (2.84 ± 1.73)	13 (20.97)	31 (50.00)	18 (29.03)	
Chronic diseases					*p >* 0.05
No	127 (2.99 ± 1.82)	29 (22.83)	58 (45.67)	40 (31.50)	
1	166 (3.23 ± 1.87)	29 (17.47)	71 (42.77)	66 (39.76)	
≥2	217 (3.11 ± 1.77)	39 (17.97)	103 (47.47)	75 (34.56)	
Self-rated health					*p* < 0.001
Very good/good	187 (3.57 ± 1.98)	26 (13.90)	81 (43.32)	80 (42.78)	
Average	232 (3.16 ± 1.69)	42 (18.10)	98 (42.24)	92 (39.66)	
Poor/very poor	91 (2.10 ± 1.32)	29 (31.87)	53 (58.24)	9 (9.89)	
Total	510 (2.16 ± 0.72)	97 (19.02)	232 (45.49)	181 (35.49)	

### 3.2 Regressions of participation in leisure activities

Participation in “at least two activities” was strongly associated with education and residence. Additional controls for self-rated health and chronic diseases confirmed the associations indicating that health was independently related to participation in at least two activities. People with less participation showed lower levels of education. Additional analyses showed no significant interaction between age, gender, marital status, living arrangements, and health (Table 4).

**Table 4:** Odds ratio and 95% confidence interval of participation in “at least two activities” in relation to demographic and health factors.

Variables	OR			
	Model 1		Model 2	
Age	0.98	(0.95, 1.01)	0.98	(0.95, 1.01)
Men	1.27	(0.73, 2.23)	1.46	(0.82, 2.59)
Education				
Primary school	1.63	(0.90, 2.95)	1.67	(0.91, 3.06)
Middle school and higher	4.58^***^	(2.01, 10.43)	4.63^***^	(2.01, 10.69)
Marital status				
Married	1.39	(0.76, 2.54)	1.45	(0.78, 2.70)
Living arrangement				
Alone	1.26	(0.60, 2.66)	1.31	(0.61, 2.82)
Income level				
≥1,000 yuan	0.80	(0.40, 1.59)	0.64	(0.31, 1.32)
Place of residence				
Urban	2.36^**^	(1.18, 4.74)	2.40^**^	(1.18, 4.86)
Self-rated health				
Average			2.52^***^	(1.33, 4.79)
Very good/good			3.36^***^	(1.53, 7.36)
Chronic diseases				
1			2.29^**^	(1.17, 4.49)
≥2			3.04^**^	(1.49, 6.18)
Constant	4.62	(0.42, 51.13)	1.09	(0.08, 14.17)

^***^ *p* < 0.01, ^**^ *p* < 0.05.

Given the possible association between the type of participation in activities and health, the association between different activities and health indicators was reexamined. Although the association between leisure activities and age did not differ, it is interesting to note that with increasing age, physical activities tend to decrease. Engaging in more types of activities was associated with a higher OR for self-rated health after adjusting for the same demographics. It remained significant for mental activities, physical activities, productive activities, and recreational activities, but not for social activities. As expected, participants living in urban areas with better self-rated health and higher educational levels were more active in mental, physical, and productive activities, and those with good self-rated health also participated in recreational activities. Additionally, married people engaged in more mental and recreational activities than those who were single, divorced, and widowed. In summary, the findings of Table 5 show the unique relation of each of the studied factors to the specific activity types. Participation patterns differed by activity type: social and productive activities showed significant variation with chronic disease status, while physical activity participation correlated with age groups.

**Table 5:** Odds ratio and 95% confidence interval (CI) of participation in the five activity types in relation to demographic and health factors.

variables	Mental	Physical	Social	Productive	Recreational
Age	1.00 (0.96, 1.03)	0.97^**^ (0.94, 1.00)	1.02 (0.99, 1.04)	0.98 (0.95, 1.01)	0.96 (0.90, 1.03)
Men	1.25 (0.75, 2.08)	0.65 (0.40, 1.04)	1.16 (0.77, 1.77)	0.76 (0.47, 1.22)	2.45 (0.60, 10.02)
Education					
Primary school	2.68 (1.13, 6.35)	1.36 (0.78, 2.39)	1.22 (0.71, 2.09)	0.80 (0.41, 1.56)	2.99 (0.67, 13.36)
Middle school and higher	17.09^***^ (7.08, 41.27)	3.20^***^ (1.64, 6.24)	1.83 (0.97, 3.44)	2.35^**^ (1.16, 4.75)	2.82 (0.48, 16.45)
Marital status					
Married	2.12^**^ (1.10, 4.07)	1.08 (0.62, 1.87)	1.22 (0.74, 2.01)	1.16 (0.66, 2.07)	3.89^**^ (1.05, 14.39)
Living arrangement					
Alone	1.37 (0.59, 3.17)	1.26 (0.63, 2.51)	1.03^**^ (0.56, 1.89)	1.06 (0.51, 2.19)	0.77 (0.21, 2.87)
Income level					
≥1,000 yuan	2.00^**^ (1.12, 3.58)	1.04 (0.59, 1.82)	1.00 (0.59, 1.69)	0.83 (0.46, 1.49)	0.23 (0.04, 1.46)
Place of residence					
Urban	1.84^**^ (1.01, 3.37)	2.31^**^ (1.32, 4.04)	0.39 (0.23, 0.66)	2.53^**^ (1.42, 4.53)	2.78 (0.55, 14.00)
Self-rated health					
Average	2.06 (0.99, 4.30)	6.12^***^ (3.34, 11.21)	1.17 (0.69, 1.99)	5.83^***^ (2.48, 13.68)	2.17 (0.61, 7.68)
Very good/good	2.53^**^ (1.14, 5.62)	6.85^***^ (3.49, 13.48)	1.40 (0.77, 2.55)	6.15^***^ (2.51, 15.08)	8.29^**^ (1.13, 60.86)
Chronic diseases					
1	1.32 (0.70, 2.49)	1.61 (0.94, 2.75)	1.65 (0.99, 2.76)	1.81^**^ (1.02, 3.21)	1.57 (0.32, 7.69)
≥2	1.31 (0.68, 2.54)	2.08^**^ (1.17, 3.69)	1.73^**^ (1.02, 2.96)	1.67 (0.93, 3.02)	3.60 (0.66, 19.69)
Constant	0.02^***^ (0.00, 0.26)	0.73 (0.08, 6.69)	0.10^**^ (0.01, 0.82)	0.10 (0.01, 1.15)	26.61 (0.13, 5273.37)

^***^ *p* < 0.01, ^**^ *p* < 0.05.

## 4 Discussion

This study found strong associations between health and leisure activities in a population-based sample of Fujian’s older adults. The major findings from the study can be summarized as follows: (1) Despite their advanced age, the majority of the population was active; (2) Watching TV/ listening to the radio was the most popular leisure activity, and recreational activities in general were the most popular type of activity; (3) average or good self-rated health, chronic diseases, and living in urban areas were associated with higher engagement in at least two activities; and (4) Health indicators were related differently to the five activity types.

According to our results, people mostly participated in watching TV. However, a previous study had proved that the longer older adults stayed before TV, the lower their cognition was, and 3.5 h per day for television viewing is the maximum (34). Compared to rural older adults, urban older adults engaged in many more leisure activities. It can be speculated that the urban lifestyle has expanded older adults’ social networks by providing accessible venues, such as libraries, community centers, and public plazas, for social interaction and physical exercise (35). These findings suggest that older adults in Fujian predominantly engage in sedentary, cognitively passive behaviors (e.g., television watching), indicating insufficient overall physical and mental activity levels. To address these disparities, policymakers should prioritize establishing community-based activity centers in rural areas and promote structured programs that integrate both physical and cognitive exercises tailored to older adults.

Older adults in advanced age groups were less engaged in physical activities, and participation in such activities requires a significant time investment relative to their leisure time allocation. This is because physical activities demand substantial somatic energy, and the oldest-old population may face age-related barriers to participation. Participants with a limited income level participated in less mental activity. A single status was associated with less participation in mentally stimulating activities, which is consistent with the recreational type. Other previous studies have shown that leisure activities vary with age (36, 37). Thus, there is a need to encourage older adults to undertake regular physical activity to achieve chronic disease control and improve their self-rated health (38).

Our findings are consistent with previous studies, which have found that that the majority of older people live an engaged lifestyle and participate in a variety of activities and that health status and chronic diseases account for a significant amount of the variance in activity engagement (39, 40). After controlling for demographic factors, self-rated health was significantly associated with leisure activity engagement levels, with older adults reporting better health status participating more than those reporting poorer health.

We observed that older adults with a middle school or higher education level were involved in three or more activities, while the subgroup with a primary school or no formal education level was inactive. The relationship between increasing activity and a higher education level was independent of the demographic factors. We presume that a lower educational level as an indicator of less knowledge of health, lower socio-economic status, and financial means may lead to a more restricted choice of leisure activities (41, 42).

Despite this study using cross-sectional data, which could not yield the complete evaluation of causal inferences, this study explored the cross-sectional association between these variables. This study was limited to a single geographical area, Fujian Province, and therefore its findings are not generalizable to the whole country. Regarding the dependent outcome, the time spent on different activities was not observed to investigate the leisure activities per day for older adults and the domain activities per day for leisure time. Another factor influencing the dependent variable is that leisure activities are based on self-selection, in which there was possible bias uncontrolled.

In summary, this study contributes to the paucity of research on evaluating the relationship between leisure activities and health factors. Self-rated health and geographic areas should be taken into account when promoting leisure activities. These results suggest that community-based senior services could leverage leisure activities as a dual-purpose intervention, enhancing recreational participation and increasing physical activity levels in older adults.

## 5 Conclusion

This study found evidence that engaging in more leisure activities is linked to better health status among older adults in China, even after adjusting for gender, age, education level, marital status, and income level. Robust public policies are warranted to promote regular leisure activity participation among older adults, which would yield significant benefits for public health and enhance the wellbeing of this population group.

## Data Availability

The data analyzed in this study is subject to the following licenses/restrictions: the data that support the findings of this study are available from Fujian Committee on Aging. Restrictions apply to the availability of these data, which were used under license for this study. Data are available from the first author with the permission of Fujian Committee on Aging. Requests to access these datasets should be directed to Zhen Li, hlzhb26@163.com.
